# TGF-β-induced growth inhibition in B-cell lymphoma correlates with Smad1/5 signalling and constitutively active p38 MAPK

**DOI:** 10.1186/1471-2172-11-57

**Published:** 2010-11-23

**Authors:** Maren Bakkebø, Kanutte Huse, Vera I Hilden, Erlend B Smeland, Morten P Oksvold

**Affiliations:** 1 Dep. of Immunology, Institute for Cancer Research, Oslo University Hospital Montebello, Oslo, Norway; 2 Centre for Cancer Biomedicine, Faculty of Medicine, University of Oslo, Oslo, Norway

## Abstract

**Background:**

Cytokines of the transforming growth factor β (TGF-β) superfamily exert effects on proliferation, apoptosis and differentiation in various cell types. Cancer cells frequently acquire resistance to the anti-proliferative signals of TGF-β, which can be due to mutations in proteins of the signalling cascade. We compared the TGF-β-related signalling properties in B-cell lymphoma cell lines that were sensitive or resistant to TGF-β-induced anti-proliferative effects.

**Results:**

TGF-β sensitive cell lines expressed higher cell surface levels of the activin receptor-like kinase 5 (Alk-5), a TGF-β receptor type 1. The expression levels of the other TGF-β and bone morphogenetic protein receptors were comparable in the different cell lines. TGF-β-induced phosphorylation of Smad2 was similar in TGF-β sensitive and resistant cell lines. In contrast, activation of Smad1/5 was restricted to cells that were sensitive to growth inhibition by TGF-β. Moreover, with activin A we detected limited anti-proliferative effects, strong phosphorylation of Smad2, but no Smad1/5 phosphorylation. Up-regulation of the TGF-β target genes Id1 and Pai-1 was identified in the TGF-β sensitive cell lines. Constitutive phosphorylation of MAPK p38 was restricted to the TGF-β sensitive cell lines. Inhibition of p38 MAPK led to reduced sensitivity to TGF-β.

**Conclusions:**

We suggest that phosphorylation of Smad1/5 is important for the anti-proliferative effects of TGF-β in B-cell lymphoma. Alk-5 was highly expressed in the sensitive cell lines, and might be important for signalling through Smad1/5. Our results indicate a role for p38 MAPK in the regulation of TGF-β-induced anti-proliferative effects.

## Background

The members of the TGF-β superfamily of cytokines, which consists of TGF-βs, bone morphogenetic proteins (BMPs) and activins, exert potent effects on proliferation, apoptosis and differentiation on many different cell types, including primary B cells [1,2]. The signalling is initiated through heterotetrameric complexes of type I and type II receptors. The cytokines bind to a type II receptor, and type I is recruited and activated through phosphorylation. There are five type II and seven type I receptors which form complexes with the TGF-β superfamily of cytokines. TGF-β induces signalling through TGF-β receptor type II (TβRII) and Alk-5 (type I), whereas activin A and B induce signalling through activin receptor type II (ActRII), activin receptor type II b (ActRIIb), Alk-4 and Alk-7 (type I) [3]. The intracellular receptor regulated Smad proteins (R-Smads) are phosphorylated by the type I receptors. Smad2 and 3 are the main R-Smads involved in TGF-β and activin signalling [4]; although several recent reports have shown that TGF-β can induce Smad1/5/8 signalling as well [5,6]. BMPs activate Smad1/5/8. R-Smads interact with the common Smad, Smad4, and translocate to the nucleus, where the complex, together with other transcription factors, regulates gene expression of e.g. Pai-1. Pai-1 plays an important role throughout many cell systems, and is involved in cell motility, angiogenesis and cancer progression [7] in addition to anti-proliferative activity [8]. It has been shown that inhibitory Smads, Smad6 and 7, inhibit the pathway at several levels, i.e. interaction between R-Smad and receptor or between R-Smads and Smad4 [3]. There is extensive crosstalk with other signalling pathways, such as p38, ERK1/2, JNK, PI3K and Wnt [9]. It is suggested that this regulation often occurs through phosphorylation of the linker region of R-Smads, which can be activating or inhibitory to the effects of TGF-βs, activins or BMPs.

In cancer, TGF-β frequently loses its anti-proliferative effects, and sometimes gains pro-proliferative features, often associated with epithelial-to-mesenchymal-transition and metastasis of epithelial cells. Loss of anti-proliferative effects can be due to mutations, gene silencing or over-expression of inhibitors [10,11]. In lymphoma and other haematological malignancies, aberrant expression of receptors and mutations in Smads have been found, although the reported frequencies of aberrations involving the TGF-β pathway in lymphoma are lower than in many other cancer types [12,13]. For example, down-regulation of TβRII RNA has been demonstrated in Burkitt lymphoma (BL) cell lines which express the full range of latent EBV genes [14].

Our aim was to elucidate the effects of TGF-β and activin A on lymphoma cell lines, to study the signalling pathways involved and to look for possible mechanisms behind sensitivity or resistance to these cytokines. We suggest that signalling through Smad1/5 can be important for maintaining sensitivity to TGF-β growth inhibitory effects. In addition, constitutively active p38 MAPK indicates a role for this kinase in the regulation of TGF-β-induced anti-proliferative effects.

## Results

### B-cell lymphoma show reduced sensitivity to TGF-β compared to primary B cells

**Figure 1:**
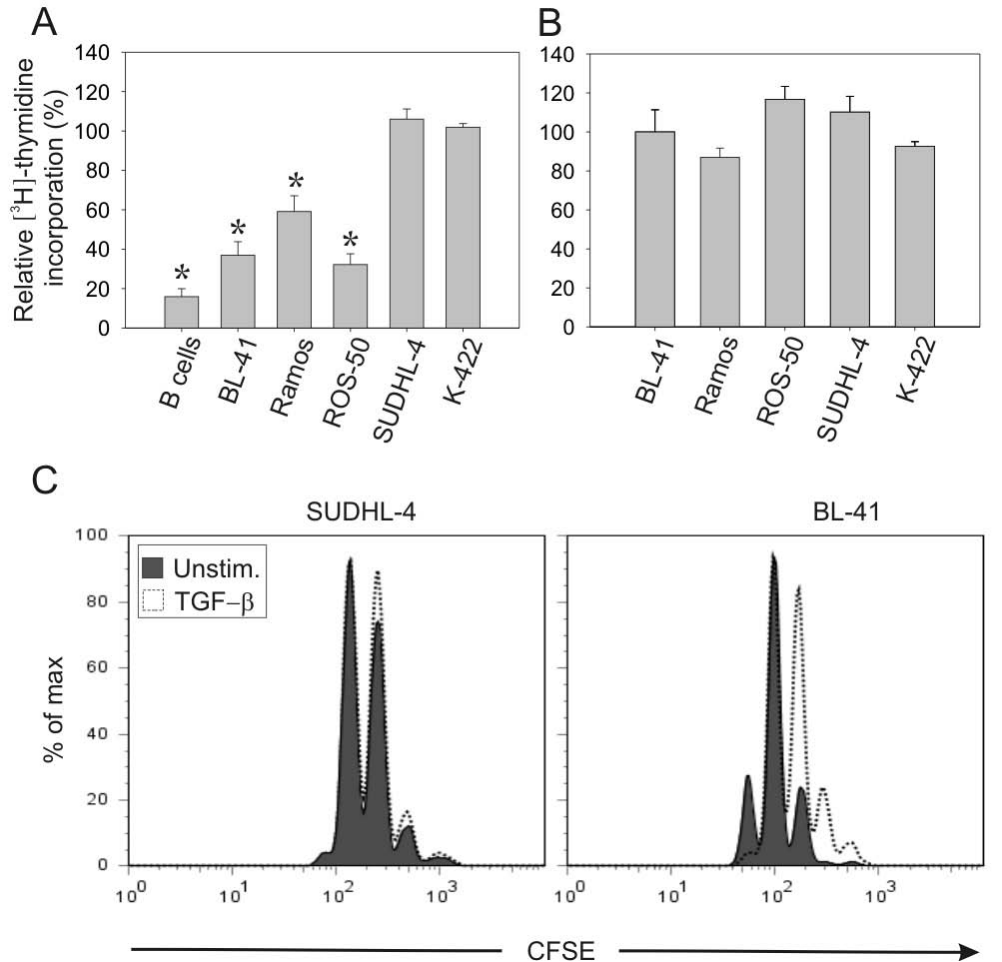
**B-cell lymphoma cell lines show reduced sensitivity to growth inhibition by TGF-β**. B-cell lymphoma cell lines and primary B cells were treated with or without TGF-β (A) and B-cell lymphoma cell lines were treated with or without activin A (B) and [^3^H]-thymidine incorporation was determined after 72 h. [^3^H]-thymidine was added for the last 4 or 16 h, for cell lines and primary cells, respectively. Depicted is relative [^3^H]-thymidine incorporation in percentage, compared to controls of each cell type (mean ± SEM, *n *= 6 (A), *n *= 3 - 4 (B)). Treated vs. control groups (A) were subjected to Wilcoxon non-parametric test. TGF-β-treated cells significantly different from the control group are indicated by asterisk (*), *p *< 0.05. (C) SUDHL-4 and BL-41 cells were labelled with 5 μM CFSE for 10 min and grown for three days with and without TGF-β. CFSE staining intensity was measured by flow cytometry. The data is representative of three similar experiments.

Many cancer types develop resistance to TGF-β-induced growth inhibition. We tested the anti-proliferative effects of TGF-β on 11 different B-cell lymphoma cell lines, and compared these results to human peripheral blood CD19^+ ^B cells. For further studies on signalling we selected five of these cell lines; three of these showed high sensitivity to TGF-β treatment; although not to the same extent as primary B cells, whereas two were resistant to the growth inhibiting effects of TGF-β (Figure 1A). In line with previously published data, TGF-β treatment of primary B cells inhibited proliferation by 85% compared to non-treated control B cells (Figure 1A). More data on additional cell lines are included in Additional file 1, Fig. S1 (two sensitive cell lines, Oci-Ly 3 and Oci-Ly 10, and one resistant cell line, Raji).

In addition to TGF-β, we tested the anti-proliferative effects of activin A and B, and detected no major effects on proliferation by these cytokines (Figure 1B and data not shown). Primary B cells were partly inhibited by activin A, with a mean inhibition of 34% (*n *= 3, data not shown).

Additionally, we measured cell division to compare the effects of TGF-β in sensitive and resistant cells. In the resistant SUDHL-4 cells no inhibition of cell division was detected. In contrast, TGF-β induced a clear inhibition in BL-41 cells after three days, as evidenced by the CFSE histograms (Figure 1C).

### TGF-β sensitive cell lines express high cell surface levels of Alk-5

**Figure 2:**
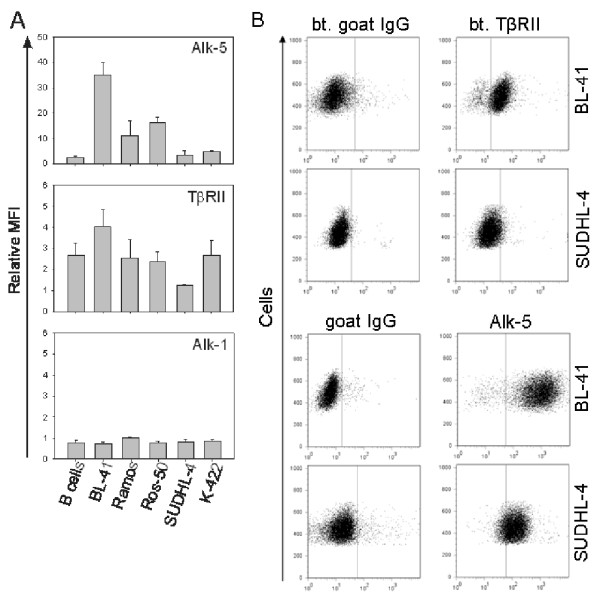
**Expression of cell surface TGF-β receptors**. Endogenous levels of the receptors Alk-5, TβRII and Alk-1 were determined by flow cytometry. Illustrated is (A) median fluorescence intensity relative to irrelevant control for primary B cells and five cell lines (mean ± SEM, *n *= 3) and (B) dot plot for BL-41 and SUDHL-4 (one representative of three). For Alk-1 and TβRII, biotinylated goat IgG is used as control, for Alk-5, goat IgG is used as control.

To determine the role of the different TGF-β receptors during Smad signalling in B-cell lymphoma, we measured endogenous cell surface levels of the receptors Alk-1, Alk-5 and TβRII by flow cytometry on lymphoma cell lines and primary B cells. The TGF-β sensitive cell lines expressed higher levels of Alk-5 compared to the resistant cell lines and primary B cells (Figure 2A and 2B). The specificity of the anti-Alk-5 antibody was tested by blocking with the peptide used for immunization before flow cytometry (data not shown). TβRII was expressed in all cell lines tested and in primary B cells, with no striking differences between TGF-β sensitive and resistant cell lines (Figure 2A and 2B). Alk-1 was expressed at low levels (Figure 2A). Furthermore, the type I and type II activin receptors (Alk-4, Alk-7, ActRII and ActRIIb) were similarly expressed in all cell lines (data not shown). It has been shown that TGF-β can signal through the BMP-receptors Alk-2 and Alk-3 [5]. We therefore examined the expression levels of these two BMP type I receptors. Of the sensitive cell lines, only ROS-50 expressed low levels of Alk-2 and Alk-3, whereas Ramos expressed some Alk-2 and higher levels of Alk-3 (Huse, K. *et al.*, submitted).

### Activation of Smad1/5 in TGF-β sensitive cells

**Figure 3:**
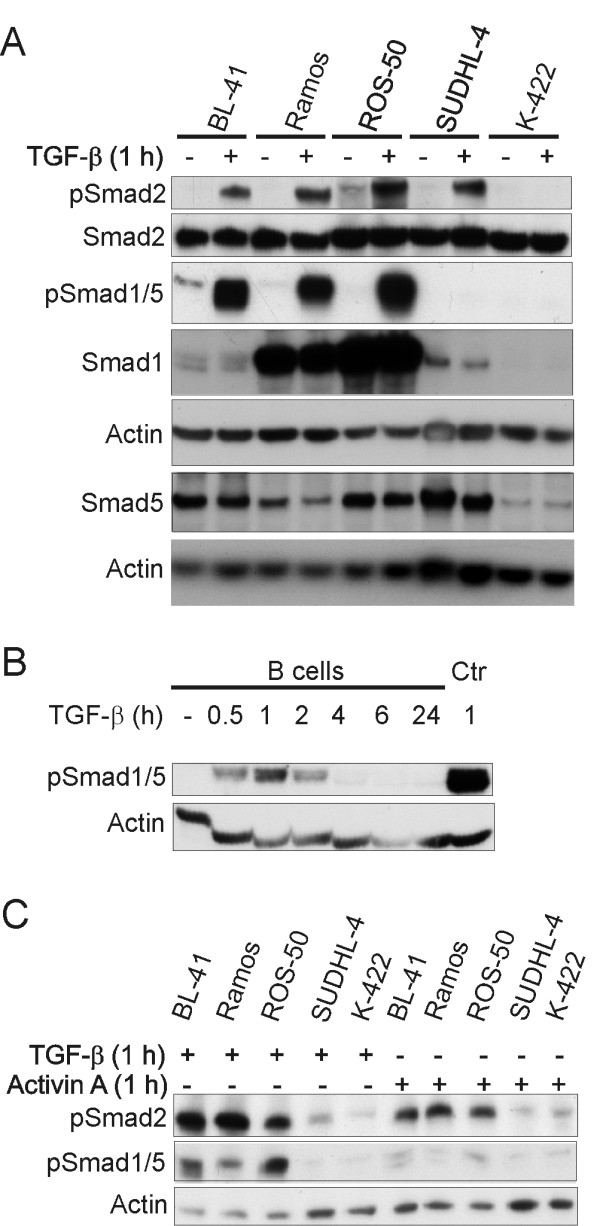
**TGF-β sensitive cell lines signal through Smad1/5 in addition to Smad2**. Cell lines were stimulated with or without TGF-β (A) and with activin A or TGF-β (C) for 1 h, and primary B cells were stimulated with or without TGF-β for 30 min, 1, 2, 4, 6 and 24 h (B), lysed, and subjected to western immunoblotting analysis, with the indicated primary antibodies. Staining with an anti-phospho-Smad1/5 antibody was applied to confirm that Smad8 was not involved (data not shown). The positive control (Ctr) in Fig. 3B is BL-41 total cell lysate. Presented is one representative blot out of three with one representative actin loading control.

To investigate signalling pathways triggered by TGF-β, Western immunoblotting analysis was performed. TGF-β induced activation of the canonical Smad2 pathway in primary B cells (data not shown) and in all cell lines, except K-422 (Figure 3A, Additional file 2, Fig. S2 and Additional file 3, Fig. S3). However, we detected no major differences in levels of Smad2 phosphorylation between sensitive and resistant cell lines. Recently, there has been focus on TGF-β signalling through Smad1/5 in addition to Smad2/3 [5,6]. Interestingly, in the sensitive cell lines as well as in primary B cells, TGF-β induced Smad1/5 phosphorylation (Figure 3A and 3B and Additional file 2, Fig. S2). Immunoblotting with anti-pSmad1/5/8 and anti-pSmad1/5 was comparable, indicating that Smad8 is not important in TGF-β signalling in B-cell lymphoma (data not shown). Activin A, which had limited effects on proliferation, induced phosphorylation of Smad2 only in the TGF-β sensitive cell lines. Phosphorylation of Smad1/5 was not detected after activin A treatment (Figure 3C). We examined endogenous levels of Smad1 and Smad2 proteins, and found that Ramos and ROS-50 cells expressed higher levels of Smad1 compared to the other cell lines. No major differences in Smad2 levels were observed (Figure 3A). Taken together, the data suggest that Smad1/5 is involved in controlling the anti-proliferative effects of TGF-β in B-cell lymphoma cell lines.

To check whether inhibitory Smads play a role in resistance to TGF-β, we assessed the endogenous protein levels of Smad6 and 7. However, only minor differences in expression levels were seen when comparing the different cell lines (data not shown).

### Activation of TGF-β target genes

**Figure 4:**
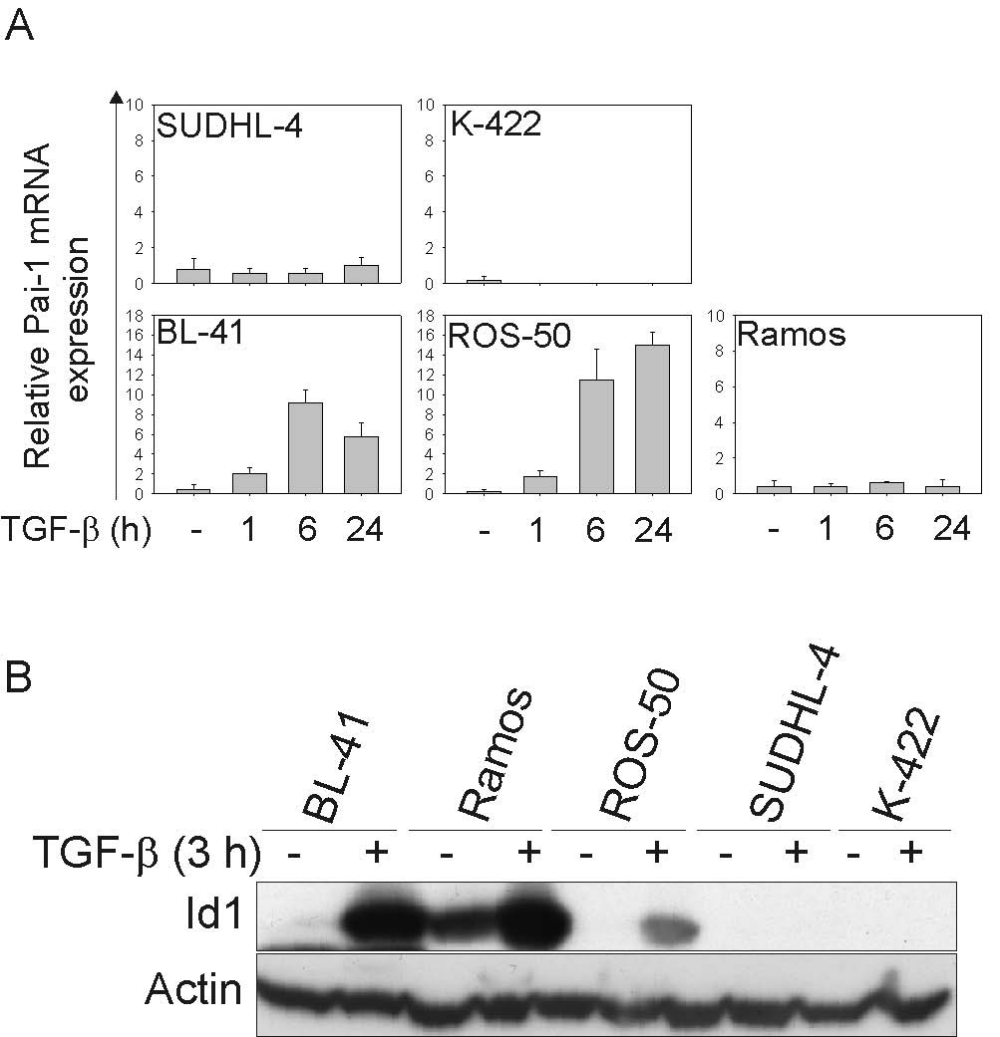
**Pai-1 and Id1 are up-regulated upon TGF-β treatment**. (A) Cell lines were stimulated with or without TGF-β for 1, 6 or 24 h, before total RNA was isolated, cDNA was synthesized and real-time RT-PCR was conducted with Pai-1 as target gene. Illustrated is relative Pai-1 mRNA expression (one representative experiment of three, ± SD). (B) Cells were stimulated without or with TGF-β for 3 h, lysed, and subjected to western immunoblotting analysis, with anti-Id1 Ab, and anti-actin Ab as loading control. Shown is one representative blot out of three.

To investigate whether the TGF-β-induced signalling continued into the nucleus and up-regulated known TGF-β target genes, we measured Pai-1 mRNA. Interestingly, TGF-β induced up-regulation of Pai-1 in two of the sensitive cell lines (Figure 4A). In addition, we demonstrated that Id1, a known BMP target gene, was induced to different degrees upon TGF-β treatment in the sensitive cell lines (Figure 4B). The resistant cell lines showed no up-regulation of either of these target genes (Figure 4A and 4B). These data imply that there are differences between TGF-β sensitive and resistant cell lines regarding induction of TGF-β target genes.

### p38 MAPK is constitutive active in TGF-β sensitive cells

**Figure 5:**
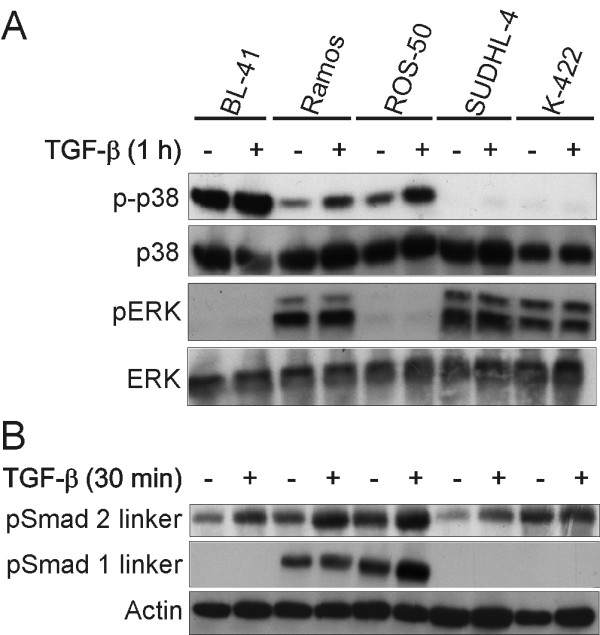
**Expression of phosphorylated ERK1/2 and p38 MAPK**. Cells were stimulated with or without TGF-β for 1 h (A) or 30 min (B), lysed, and subjected to western immunoblotting analysis with the indicated primary antibodies. Shown is one representative blot out of three.

We further investigated other signalling pathways known to crosstalk with the canonical Smad pathway. Of interest, we found high constitutive levels of phosphorylated p38 MAPK (Thr180/Tyr182) in the TGF-β sensitive cell lines (Figure 5A and Additional file 4, Fig. S4). The resistant cell lines expressed minimal levels of active p38 MAPK compared to the sensitive cell lines. We also found high constitutive levels of phosphorylated ERK1/2 MAPK (Thr202/Tyr204) in the TGF-β resistant cell lines, but also in one of the sensitive cell lines (Figure 5A). TGF-β did not affect the level of phosphorylated ERK1/2. Screening of other activated signalling molecules, i.e. phosphorylated Akt, JNK MAPK, TAK and MKK 3/6 did not reveal any correlation to sensitivity or resistance to TGF-β (data not shown).

Due to high levels of activated ERK1/2 MAPK in the resistant cell lines, and the fact that this can alter the canonical Smad signalling pathway through phosphorylation of the linker region, we investigated phosphorylation levels of the Smad2 (Ser245/Ser250/Ser255) and Smad1 (Ser206) linker regions. Smad1 linker phosphorylation was detectable in two TGF-β sensitive cell lines, and TGF-β only slightly altered the level of linker phosphorylation in these cell lines (Figure 5B). In contrast, no major differences in Smad2 linker region phosphorylation were observed between the sensitive and resistant cell lines. These results imply that activated ERK1/2 MAPK could be involved in resistance to TGF-β in B-cell lymphoma cell lines, although phosphorylation of the linker region of Smad2 seems not to be the mechanism. We suggest that activated p38 MAPK could be important for sensitivity to TGF-β.

### Inhibition of p38 MAPK leads to reduced sensitivity to TGF-β

**Figure 6:**
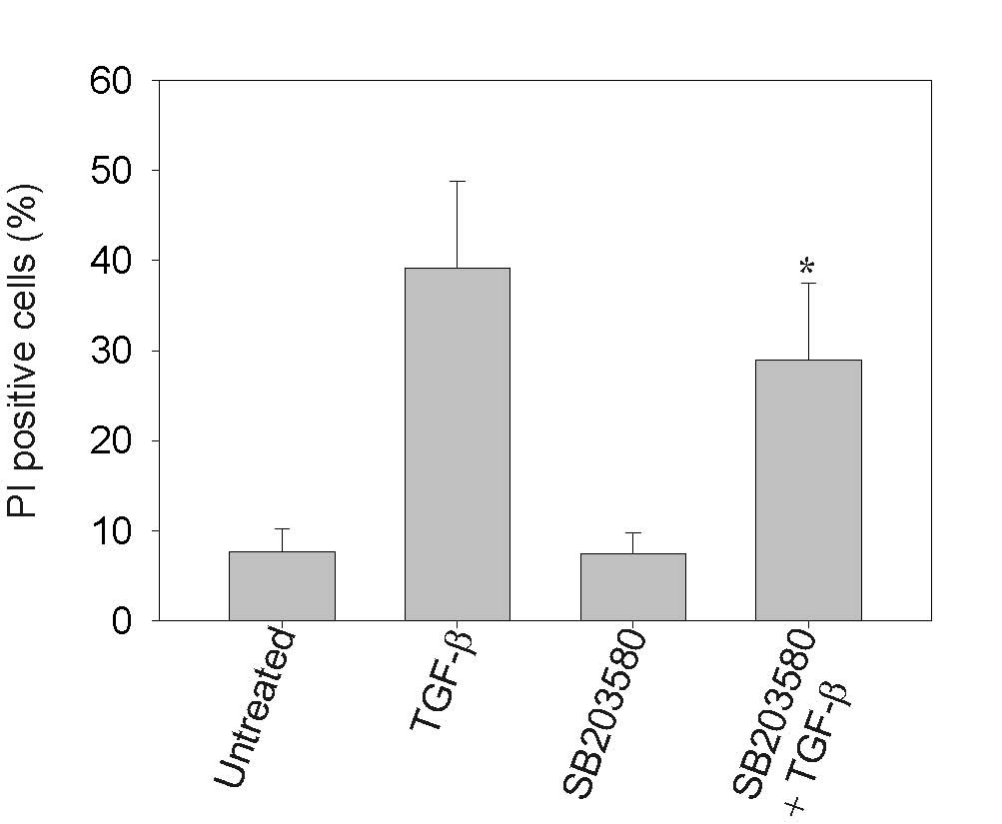
**Inhibition of p38 MAPK leads to less TGF-β-induced cell death**. Ramos cells were treated with or without the p38 MAPK specific inhibitor SB203580 for 1 h, followed by incubation with or without TGF-β, stained with PI after 72 h and analyzed by flow cytometry. Presented is percentage of PI-positive cells (mean ± SEM, *n *= 6). Treatment with p38 MAPK inhibitor and TGF-β together were subjected to Wilcoxon non-parametric test against treatment with TGF-β alone. Significance is indicated with asterisk (*), *p *< 0.05.

To test whether p38 contributes to TGF-β sensitivity, we used the p38-specific inhibitor SB203580 in the TGF-β sensitive cell line Ramos. When phosphorylation of p38 was inhibited, we observed reduced sensitivity to TGF-β-induced anti-proliferative effects compared to the control group (Figure 6). TGF-β induced cell death in 39% of the cells, whereas TGF-β together with SB203580 differed significantly with 29% cell death (*p *< 0.05, Figure 6). The p38 inhibitor also reduced TGF-β-induced apoptosis as determined by TUNEL analysis (data not shown). Inhibition of ERK1/2 MAPK did not alter the effects of TGF-β on the resistant cell lines (data not shown). Thus, inhibition of p38 MAPK partially counteracts TGF-β-induced growth suppression in Ramos cells, suggesting a role for p38 MAPK in the regulation of TGF-β-induced anti-proliferative effects.

## Discussion

It is known from several cancer types that TGF-β loses its anti-proliferative effects, often due to mutations in receptors or Smad proteins [15,16]. Haematological malignancies, especially B-cell lymphoma, have received less attention regarding TGF-β signalling. We sought to elucidate the effects of TGF-β on cell lines from different B-cell lymphoma subtypes, working with endogenous levels of gene expression. We found that the B-cell lymphoma cell lines examined displayed reduced sensitivity to TGF-β compared to primary B cells. This indicates that loss of sensitivity towards the growth inhibitory effects of TGF-β can be of importance for the development of B-cell lymphoma.

Although Smad2 and 3 are the main R-Smads for TGF-β signalling, we found no clear differences in TGF-β-induced Smad2 signalling when comparing sensitive and resistant cell lines. Moreover, we detected that activin A and B exerted limited anti-proliferative effects on the B-cell lymphoma cell lines, even though clear Smad2 signalling was observed in the TGF-β-sensitive cell lines upon activin A stimulation. This further indicates that Smad2 phosphorylation is not directly correlated to inhibition of proliferation. Of note, recent studies have revealed that TGF-β can also activate the Smad1/5/8 pathway. Interestingly, we observed a clear correlation between sensitivity to TGF-β and Smad1/5 phosphorylation as TGF-β induced phosphorylation of Smad1/5 in sensitive cell lines only. Smad1/5 signalling upon TGF-β treatment has to our knowledge previously not been reported in primary B cells. These data suggest that signalling through Smad1/5 is important for the functional effects of TGF-β on B-cell lymphoma cell lines of different origin. In agreement with our data, Munoz *et al. *have previously reported induction of Smad1 phosphorylation upon TGF-β treatment in follicular lymphoma cell lines and one diffuse large B-cell lymphoma cell line [17]. Moreover, they demonstrated that the functional effects of TGF-β were diminished upon treatment with Smad1 siRNA. Taken together, available data suggest that Smad1/5 is crucial for the anti-proliferative effects of TGF-β.

We found that sensitive cell lines showed higher endogenous Alk-5 levels and this expression correlated to Smad1/5 activation, as it was highly expressed in the cell lines where TGF-β induced phosphorylation of Smad1/5. Similar results have been found in other cell systems [6]. Data by Wrighton *et al*. suggest that Alk-5 has the ability to phosphorylate Smad1, and that Smad1 can co-precipitate Alk-5 in HEK293T cells. In other cell systems, additional receptors have been demonstrated to be necessary. Daly *et al*. proved that TβRII and Alk-5 were required, but not sufficient for Smad1/5 phosphorylation [5]. They found that Alk-2 or Alk-3 can co-precipitate with TβRII and Alk-5, and that forming of the receptor complexes is dependent on cell type. Among the cell lines which induced Smad1/5 signalling, only Ramos expressed some Alk-2 and higher levels of Alk-3. Alk-1 was expressed at such low levels that it is unlikely to be involved. This was expected, because Alk-1 is believed to be present only in endothelial cells [18,19]. TβRII is most likely involved in Smad2 and Smad1/5 signalling in our cell lines, as it is the only known type II receptor for TGF-β [3]. However, the TβRII expression level differed in both sensitive and resistant cell lines. Smad2 signalling upon activin A stimulation is detected in Ramos, ROS-50 and BL-41 cells. Abrogated Smad2 signalling in the other cell lines is most likely not due to reduced expression of receptors, as we detected nearly equal expression of all known activin receptors in our cell lines. Thus, Alk-5 might be the receptor which is crucial for Smad1/5 signalling and TGF-β-induced anti-proliferative effects.

Previous work has shown a correlation between activated p38 MAPK and the apoptotic effects of TGF-β in BL-41 cells [20]. In accordance with this study, we found that p38 was constitutively phosphorylated in cell lines sensitive to growth inhibition by TGF-β. In contrast, TGF-β resistant cell lines expressed high levels of phosphorylated ERK1/2 MAPK. We successfully inhibited p38 in Ramos cells, and showed that the anti-apoptotic effects of TGF-β is dependent, at least to some degree, on the activity of p38. It is possible that p38-induced sumoylation of Smad4, which enhances TGF-β and BMP target gene activation, could explain the positive effect of phosphorylated p38 on TGF-β growth inhibition, as suggested by Ohshima *et al*. [21]. Possibly, one needs to induce ERK1/2 in addition to inhibiting p38 to diminish the effects of TGF-β. Interestingly, we detected phosphorylated ERK1/2 in Ramos cells, whereas in BL-41 and ROS-50 cells this phosphorylation was not seen. This might explain why the effects of TGF-β were reduced only in Ramos cells and not in BL-41 and ROS-50 cells (data not shown) upon adding the p38 inhibitor. Phosphorylation of the R-Smad linker region may inhibit translocation of activated Smad-complexes to the nucleus. It is demonstrated that ERK1/2 phosphorylates the linker region of Smad1 and Smad2, and this can inhibit signal transduction and the anti-proliferative effects of TGF-β [22,23]. However, the consequences of linker-phosphorylation remain controversial [24], and we did not detect any higher levels of phosphorylation of the Smad2 linker region in TGF-β resistant compared to sensitive cell lines. The Smad1 linker region was phosphorylated in Ramos and ROS-50 cells, and this might even induce Smad1/5 signalling by TGF-β in these cells.

Id1 is a known BMP-responsive gene, which is up-regulated upon Smad1/5/8 signalling [25,26]. However, TGF-β-induction of Id proteins has previously been found in a BL cell line, CA46 [27], although it was not investigated whether Smad1/5 signalling was involved. We demonstrate induction of Id1 protein in the TGF-β sensitive cell lines (BL-41, Ramos and ROS-50) after 3 h of TGF-β stimulation. Opposed to that, Daly *et al. *did not detect induction of a luciferase reporter containing two repeats of a BMP response element in cell types where TGF-β also signals through Smad1/5 [5]. Induction of Id1 is possibly dependent on the cell type. It has been reported that TGF-β represses Id expression in epithelial cells [28].

## Conclusion

To summarize, three B-cell lymphoma cell lines showed sensitivity to the TGF-β anti-proliferative effects. Sensitivity to growth inhibition by TGF-β might depend on Smad1/5 signalling in lymphoma cell lines, which possibly initiates via Alk-5 and terminates in up-regulation of Id1 and other target genes. We suggest that the regulation of proliferation by TGF-β is at least partly dependent on activated p38 MAPK. Further knock-down studies need to be assessed to confirm this theory. In the future, therapies which can restore sensitivity of lymphoma cells to TGF-β growth control by inducing Smad1/5 signalling can be helpful in treatment of B-cell lymphoma patients.

## Methods

### Cell culture

BL cell lines Ramos, BL-41 and Raji, diffuse large B-cell lymphoma cell lines of germinal centre B type SUDHL-4 and of activated B cell type Oci-Ly 3 and Oci-Ly 10 and follicular lymphoma cell lines K-422 and ROS-50 were cultured in RPMI (PAA Laboratories, Austria) with 100 Units/ml penicillin and 0.1 mg/ml streptomycin (PAA Laboratories) and 10% fetal calf serum (PAA Laboratories), except for the Oci Ly-cells, which were cultured in IMDM (Invitrogen, CA, USA) with 55 μM β-Mercaptoethanol (Invitrogen), 100 Units/ml penicillin and 0,1 mg/ml streptomycin (PAA Laboratories) and 10% human plasma (SeraCare Life Sciences, Inc., California, USA), at 37°C with 5% CO_2 _in air. Prior to all experiments, cells were grown under serum free conditions over night in X-VIVO 15 (BioWhittaker, Switzerland). Primary human B cells from peripheral blood were isolated using CD19^+ ^Dynabeads (Invitrogen), as described by Rasmussen *et al*. [29]. Peripheral blood was provided by the Blood Bank at Ullevål University Hospital with formal agreement by the blood donors, and approval by the regional ethics committee.

### Reagents

Carrier-free huTGF-β1 (10 ng/ml) and activin A and B (10 ng/ml) were purchased from R&D Systems (MN, USA). Anti-IgM (10 μg/ml) was obtained from Jackson Immuno Research (PA, USA). [^3^H]-thymidine was purchased from American Radiolabeled Chemicals (MO, USA). The following Ab were used: Anti-phospho-Smad2, -phospho-Smad1/5/8, -phospho-Smad1/5, -Smad1, -Smad2, -Smad6, -phospho-p38 MAPK, -p38 MAPK, -phospho-ERK1/2 MAPK -phospho-TAK 1, -phospho-MKK3/MKK6 and -phospho-JNK MAPK Ab (Cell Signalling Technology, MA, USA), anti-actin, -ERK MAPK and -Id1 Ab (Santa Cruz, CA, USA), anti-Smad7 Ab (Abcam, MA, USA), biotinylated anti-Alk-1, -TβRII, -ActRII and -ActRIIb Ab and anti-Alk-4 and -Alk-5 Ab (R&D systems), anti-Alk-7 Ab (Millipore, MA, USA) and HRP-coupled secondary anti-rabbit, -mouse and -goat IgG Ab (DakoCytomation, Denmark). ERK inhibitors FR180204 and UO126 and p38 inhibitor SB203580 were purchased from Calbiochem (Darmstadt, Germany). The Alk-5 peptide used for blocking of anti-Alk-5 antibody was obtained from R&D systems.

### Cell proliferation assays

Cells were harvested after 72 h using an automated cell harvester (Filtermate 196, Packard Instrument Company Inc., CT, USA), and [^3^H]-thymidine incorporation into DNA was measured on a scintillation counter (TopCount, Packard Instrument Company Inc.). Assays were performed in triplicates in round bottom 96-well plates, 200 μl per well; 0.1 × 10^6 ^cells/ml for cell lines and 0.375 × 10^6 ^cells/ml for B cells. [^3^H]-thymidine was added 4 or 16 h before measurement, respectively.

To monitor cell death, 5 μg/ml PI was used for analysis by flow cytometry (Becton Dickinson, FACSCalibur, NJ, USA). Cell death was measured for every functional experiment conducted.

For CFSE proliferation analysis cells were labeled with 5 μM CFSE (Molecular Probes, OR, USA) in PBS with 0.1% BSA for 10 min at 37°C. The labeling reaction was quenched by addition of cold PBS with 20% FCS. Cells were incubated in pre-warmed X-VIVO 15 and cultured over night before cells with identical CFSE staining intensity was sorted. The CFSE sorted cells were cultured for up to 3 days with or without TGF-β. FACS analysis allowed gating on individual CFSE generations (Becton Dickinson, FACS CantoII).

### Apoptosis assay

Cells were harvested after 3 days and stained with TUNEL (Roche, Switzerland) according to the manufacture's protocol. Cells were analyzed by flow cytometry (Becton Dickinson, FACSCalibur).

### Western immunoblotting analysis

Cells were lysed in Tris lysis buffer, pH 6.8 (62.7 mM Tris-HCl, 10% (v/v) glycerol, 2.3% (w/v) SDS, 5% β-Mercaptoethanol, 1× protease inhibitor mixture (Complete Mini, Roche) and 1× phosphatase inhibitor mixture (PhosSTOP, Roche)). Lysates were incubated at 95°C for 10 min, cleared by centrifugation at 15700 g for 5 min and protein concentrations were determined with the BioRad (CA, USA) protein assay. Samples (30-40 μg/lane) were run on 10% or 12% SDS-polyacrylamide gels (Pierce, IL, USA) or 10% Tris-HCl Criterion gels (BioRad) and transferred to PVDF membranes (Millipore). Membranes were blocked in 5% non-fat dry milk or 5% BSA (Sigma-Aldrich, MO, USA) in TBST buffer pH 7.6, according to the antibody manufacturer's protocol. PVDF membranes were incubated over night at 4°C with antibody diluted in 5% non-fat dry milk or 5% BSA in TBST buffer. HRP-conjugated anti-mouse, -goat and -rabbit IgG antibodies incubated for 60 min at room temperature were used followed by detection using ECL or ECL-plus (GE Healthcare, NJ, USA).

### Detection of cell surface receptor expression

Cells were blocked in 1 mg/ml γ-globulin (aggregated at 63°C, 20 min (Sigma-Aldrich)) for 10 min on ice, prior to staining with anti-Alk-1, -Alk-5 or -TβRII antibodies for 30 min at 4°C. Avidin-PE (BioRad) was used as second layer. Goat Ig and biotinylated Goat Ig were used as controls. Cells were washed in PBS. Receptor levels were detected by flow cytometry (Becton Dickinson, FACSCalibur and FACS CantoII).

### Real time RT-PCR analysis

RNA was isolated using the Absolutely RNA Miniprep kit (Stratagene, CA, USA) following the manufacturer's instructions. RNA concentration was obtained using NanoDrop-1000 Spectrophotometer (Thermo Fisher Scientific Inc., MA, USA), and quality was assessed by gel electrophoresis using 1.5% agarose gel. RNA was stored at -80°C. Using the TaqMan kit (Applied Biosystems, CA, USA), cDNA was obtained from 1 μg RNA for each sample and 400 ng RNA for the minus reverse transcriptase (-RT) negative controls. Conditions: 10 min at 25°C, 40 min at 42°C and 10 min at 95°C.

Real-time RT-PCR was conducted with TaqMan Universal PCR master mix (Applied Biosystems) with Pai-1 (Applied Biosystems) as the target gene and PGK-1 (Applied Biosystems) as the control gene. The cDNA used assembled a total of 10 ng RNA per reaction. The samples had a total volume of 25 μl, and were run on ABI Prism 7000 Sequence Detection System. Conditions: 2 min at 50°C, 10 min at 95°C and 40 cycles of 15 s at 95°C and 1 min at 60°C. Relative expression levels were calculated using the threshold cycle ΔΔCt-method as described by the manufacturers' protocol. Instead of calibrating the samples to a control sample, samples were calibrated to the number 10.

### Statistical analysis

Wilcoxon non-parametric test was used for all statistical measurements, with *p*-value less than 0.05 considered as significant. The statistical significance analysis in Figure 1A has been conducted on primary data, although the data depicted in the figure are normalized.

## Authors' contributions

MB participated in the design, conducted most of the experiments, and drafted the manuscript. KH conducted some of the initial experiments. VIH participated in some of the experiments. EBS participated in the study design, and obtained funding. MPO developed the project idea, participated in the study design, coordinated and conducted some of the experiments. All the authors have critically read, commented and approved the final manuscript.

## Supplementary Material

Additional file 1 **Fig. S1. B-cell lymphoma cell lines show reduced sensitivity to growth inhibition by TGF-β**. B-cell lymphoma cell lines were treated with or without TGF-β and [^3^H]-thymidine incorporation was determined after 72 h. [^3^H]-thymidine was added for the last 4 h. Depicted is relative [^3^H]-thymidine incorporation in percentage, compared to controls of each cell type (mean ± SEM, *n *= 6). Click here for file

Additional file 2 **Fig. S2. TGF-β sensitive cell lines signal through Smad1/5 in addition to Smad2**. Cell lines were stimulated with or without TGF-β for 1 h, lysed, and subjected to western immunoblotting analysis, with the indicated primary antibodies. Presented is one representative blot out of three. Actin was used as loading control. Click here for file

Additional file 3 **Fig. S3. Both TGF-β sensitive and resistant cell lines signal through Smad2**. The volume of each band from Western immunoblots with pSmad2 and actin antibodies was calculated using Quantity One Analysis Software to quantify the phosphorylation of Smad2 upon TGF-β stimulation. The measured values were normalized against actin and the relative expression in TGF-β-treated BL-41 cells. Shown is relative pSmad2 expression in both sensitive and resistant cell lines, n = 6 (mean ± SEM). Click here for file

Additional file 4 **Fig. S4. Sensitive cell lines express activated p-p38**. Cells were stimulated with or without TGF-β for 1 h, lysed and subjected to western immunoblotting analysis with p-p38 and actin as primary antibodies. Shown is one representative blot out of three, and one representative actin control. Click here for file
