# Supplementary figures and images for: TGF-β-induced growth inhibition in B-cell lymphoma correlates with Smad1/5 signalling and constitutively active p38 MAPK
Source: BMC Immunol. 2010 Nov 23;11:57. doi: 10.1186/1471-2172-11-57 (PMC3006362; doi:10.1186/1471-2172-11-57)

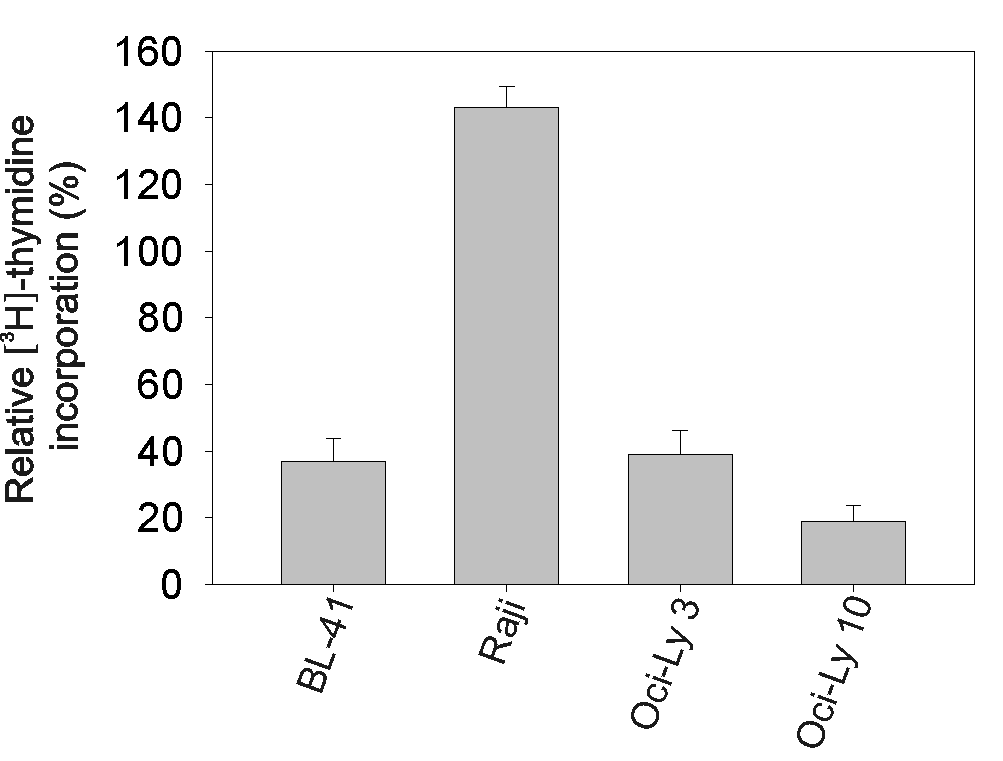

Supplement: Additional file 1 — Fig. S1. B-cell lymphoma cell lines show reduced sensitivity to growth inhibition by TGF-β. B-cell lymphoma cell lines were treated with or without TGF-β and [3H]-thymidine incorporation was determined after 72 h. [3H]-thymidine was added for the last 4 h. Depicted is relative [3H]-thymidine incorporation in percentage, compared to controls of each cell type (mean ± SEM, n = 6). [file 1471-2172-11-57-S1.TIFF]

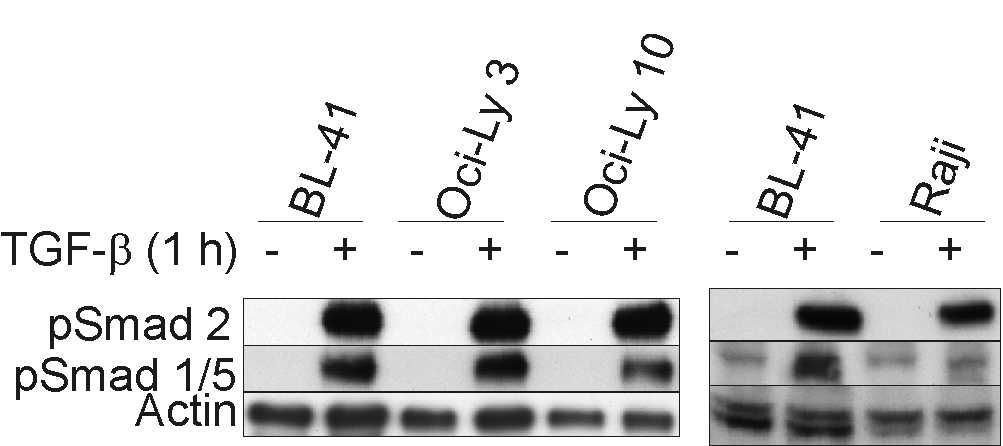

Supplement: Additional file 2 — Fig. S2. TGF-β sensitive cell lines signal through Smad1/5 in addition to Smad2. Cell lines were stimulated with or without TGF-β for 1 h, lysed, and subjected to western immunoblotting analysis, with the indicated primary antibodies. Presented is one representative blot out of three. Actin was used as loading control. [file 1471-2172-11-57-S2.TIFF]

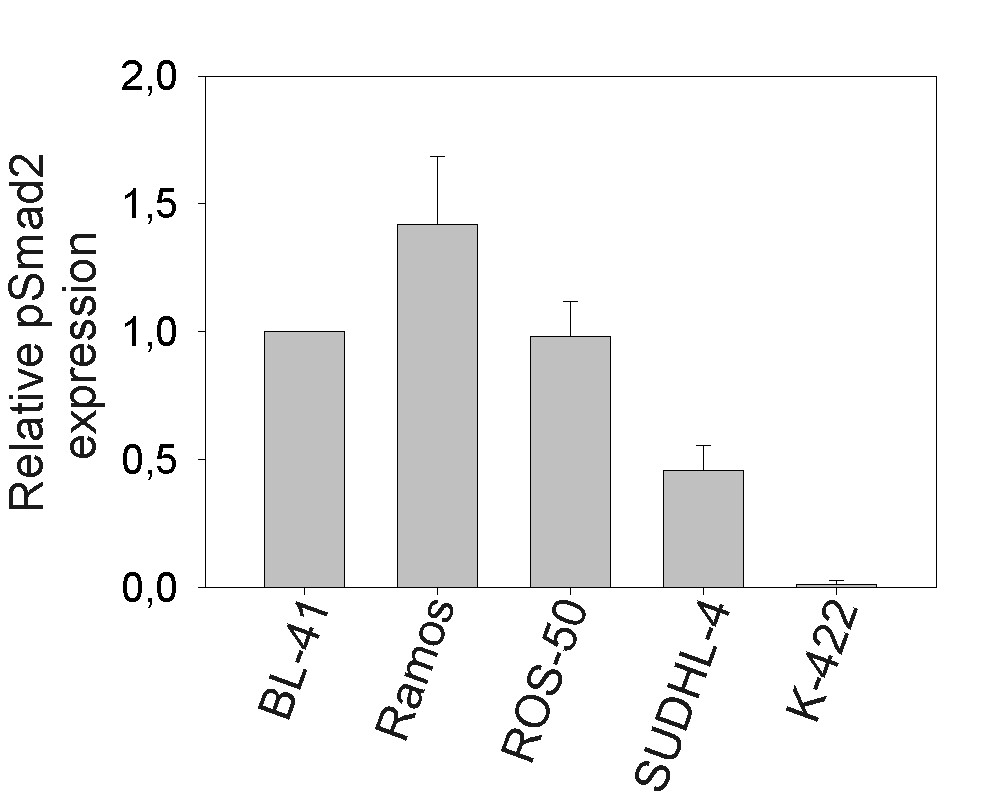

Supplement: Additional file 3 — Fig. S3. Both TGF-β sensitive and resistant cell lines signal through Smad2. The volume of each band from Western immunoblots with pSmad2 and actin antibodies was calculated using Quantity One Analysis Software to quantify the phosphorylation of Smad2 upon TGF-β stimulation. The measured values were normalized against actin and the relative expression in TGF-β-treated BL-41 cells. Shown is relative pSmad2 expression in both sensitive and resistant cell lines, n = 6 (mean ± SEM). [file 1471-2172-11-57-S3.TIFF]

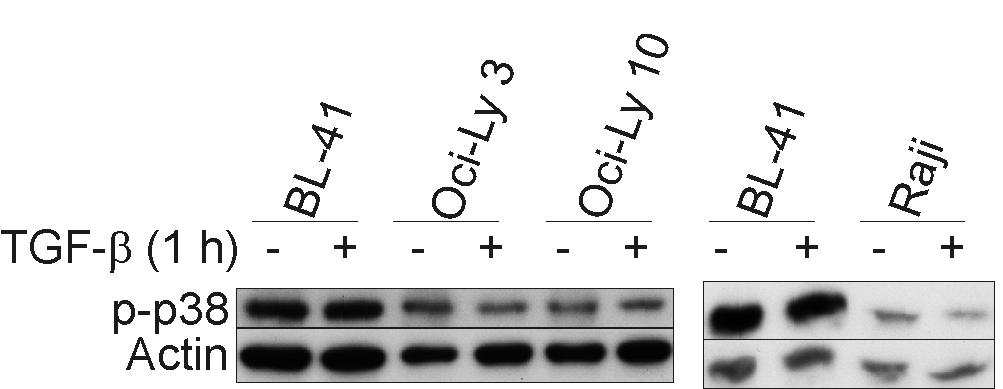

Supplement: Additional file 4 — Fig. S4. Sensitive cell lines express activated p-p38. Cells were stimulated with or without TGF-β for 1 h, lysed and subjected to western immunoblotting analysis with p-p38 and actin as primary antibodies. Shown is one representative blot out of three, and one representative actin control. [file 1471-2172-11-57-S4.TIFF]
